# First Report of *Trypanosoma* sp. in Spectacled Caiman (*Caiman crocodilus*): Morphological and Phylogenetic Relationships

**DOI:** 10.5402/2013/328794

**Published:** 2013-08-13

**Authors:** Arlei Marcili, Andrea P. da Costa, Herbert S. Soares, Igor C. L. Acosta, Julia T. R. de Lima, Antonio H. H. Minervino, Solange M. Gennari

**Affiliations:** Faculdade de Medicina Veterinária e Zootecnia, Universidade de São Paulo, Avenida Prof. Orlando Marques de Paiva 87, Cidade Universitária, 05508-270 São Paulo, SP, Brazil

## Abstract

In Crocodylidae family three trypanosomes species were described, *T. grayi* in African crocodilian and *T. cecili* and *Trypanosoma* sp. in Caimans species from Brazil. *T. grayi* was transmitted by tsetse flies and the vector of Brazilian caimans trypanosomes is unknown. We characterized first Brazilian trypanosome isolated in spectacled caiman (*Caiman crocodilus*) from Mato Grosso State in Brazil. Morphological findings in epimastigotes forms from axenic culture showed high similarity with *Trypanosoma* sp. described in *Caiman yacare* from Brazilian Pantanal. Phylogenetic studies performed with SSU rDNA and gGAPDH (glyceraldehydes-3-phosphato dehydrogenase glycosomal) clustering in *T. grayi* Clade and together to genotype Cay 01 from *Trypanosoma* unnamed species isolated in *C. yacare*. This is the first isolate of *Trypanosoma* sp. from *C. crocodilus* and the phylogenetic position with isolates in *C. yacare* from Pantanal region and demonstrates the low host specificity of cayman trypanosomes in Brazil.

## 1. Introduction

The order Crocodylia includes 23 living species and three families are recognized, Crocodylidae, Gavialidae, and Alligatoridae [1]. Most species of families Crocodylidae and Gavialidae occur in Africa and Asia. Only Alligatoridae occurs in South America and is composed of six species, *Paleosuchus palpebrosus*, *P. trigonatus, Melanosuchus niger, Caiman yacare, C. latirostris, and C. crocodilus* [2]. 

The spectacled caiman (*Caiman crocodilus*) has the widest distribution of the New World crocodilians with geographic range from southern Mexico to Peru and Brazil. This geographic variability enabled a segregation of this species into four subspecies [3, 4]. A single subspecies occur in North and Central Brazil, *Caiman crocodilus crocodilus* [5].

The crocodilians are host to a wide variety of parasites, like intestinal parasites (nematodes and trematodes) [6–10] and hemoparasites (haemogegarines and trypanosomes) [10–13].

The species of genus *Trypanosoma* are parasites of all vertebrate classes (fish, amphibians, reptiles, birds, and mammals) with life cycles alternating between vertebrates and invertebrates hosts. Most species develop in arthropod vectors, which may belong to different orders and families, while fish, amphibian, and reptiles parasites are transmitted by leeches or insects. Other species are only mechanically transmitted. This genus has several stages, present in different combinations, in blood and/or tissues in the vertebrate and invertebrate hosts [14–19].

In reptiles are described about 80 species of *Trypanosoma* parasites, including 42 in lizards, 14 in turtles, 21 in snakes, and 3 in crocodilians. The species descriptions are based on morphology of blood forms, host, and geographic origin [20]. In Africa, *T. grayi* has been described in *Osteolaemus tetraspis* and *Crocodilus niloticus,* both in family Crocodylidae [21]. In Brazil, *Trypanosoma *unnamed species was described in *Caiman yacare* [22] and *T. cecili* in *Caiman crocodilus* [13].

Phylogenetic studies performed with a large number of isolates from Africa (*T. grayi*) and Brazil (*Trypanosoma* sp.) positioned all sequences from whole SSU rDNA and gGAPDH in a unique monophyletic branch named *T. grayi* Clade [19, 23, 24]. The Brazilian isolates are segregated in two genotypes, named Cay01 and Cay02, and are different from *T. grayi* isolates [19]. The host of Brazilian trypanosomes isolates is *Caiman yacare* captured in Pantanal region.

In the present study, we described a first record of *Trypanosoma* sp. in spectacled caiman (*Caiman crocodilus*) from Mato Grosso State and addressed the phylogenetic relationships with other crocodilian trypanosomes.

## 2. Materials and Methods

### 2.1. Study Areas and Capture of Spectacled Caiman

The sample was collected from a single animal captured by ethnic Indians Tapirapé within the area of their Indian reserve in Mato Grosso State, county of Confresa (10°38′22′′ S, 51°34′08′′ W), composed of a mosaic of Amazonian Rain Forest and Cerrado biomes. The animal included in this study was obtained from legal hunting activity, authorized by IBAMA, held by the indigenous population. Three specimens of *C. crocodilus*, after being handed and immobilized, were anaesthetized and the blood samples and tissue samples (spleen, heart, kidney, lung, and liver) were collected.

### 2.2. Isolation in Culture of *Trypanosoma *sp. from Spectacled Caiman

For *Trypanosoma* isolation, blood samples from spectacled caiman (*Caiman crocodilus*) were inoculated in vacutainer tubes with a biphasic medium containing 15% of sheep red blood cells with 4% Blood Agar Base and overlaid with liquid LIT medium supplemented with 20% of FBS as before [19]. The culture was incubated at 28°C and expanded in LIT (Liver Infusion Tryptose) medium for DNA preparation. The isolate (CBT 02) was cryopreserved in liquid nitrogen in the Department of Preventive Veterinary Medicine and Animal Health, Faculty of Veterinary Medicine, University of São Paulo. Samples from cultures were smeared on glass slides and fixed with methanol and stained with Giemsa and photographed.

### 2.3. Molecular Study

DNA sample was extracted from parasite mass of trypanosome culture using the phenol-chloroform method and primary samples were purified using the Wizard DNA Clean-Up System (Promega, Fitchburg, Wisconsin). Extracted DNA samples were subjected to conventional polymerase chain reaction (PCR) targeting a fragment of approximately 900 base pairs (bp) of the V7V8 SSU rDNA [18, 19] and approximately 800 bp of the gGAPDH (glyceraldehydes-3-phosphate dehydrogenase glycosomal) as previously described [23]. PCR products of the expected sizes were purified and sequenced in an automatic sequencer (Applied Biosystems/PerkinElmer, model ABI Prism 310 Genetic, Foster City, CA) according to the manufacturer's recommendations. The nucleotide sequences generated were deposited in GenBank under the accession numbers JQ768791 and JQ768792, respectively, for V7V8 SSU rDNA and gGAPDH genes. These sequences were concatenated and aligned using ClustalX [25] and adjusted manually using GeneDoc software [26] with sequences previously determined from other trypanosomatids species available in Genbank (Table 1). The V7V8 region of SSU rDNA of crocodilian isolates was used to construct a dendogram for intraspecific analysis. The phylogenetic tree was inferred by the maximum parsimony (MP) method using PAUP version 4.0b10 [27] with 500 replicates of random addition taxa and TBR branch swapping. Bayesian analysis (B) was performed by Mrs. Bayes v3.1.2 [28] and 1.000.000 generations were employed as using GTR substitution model and four categories range proportion of invariant sites. Posteriori probabilities were used to support branches.

## 3. Results

The positive hemoculture obtained from a single spectacled caiman captured generated one isolate maintained in culture (33%). Cultures were maintained in axenic medium LIT and used for morphological analysis. Unfortunately, no smears were obtained from the blood of the spectacled caiman. All tissues, including the positive animal, were negative to trypanosome DNA barcoding.

**Figure 1:**
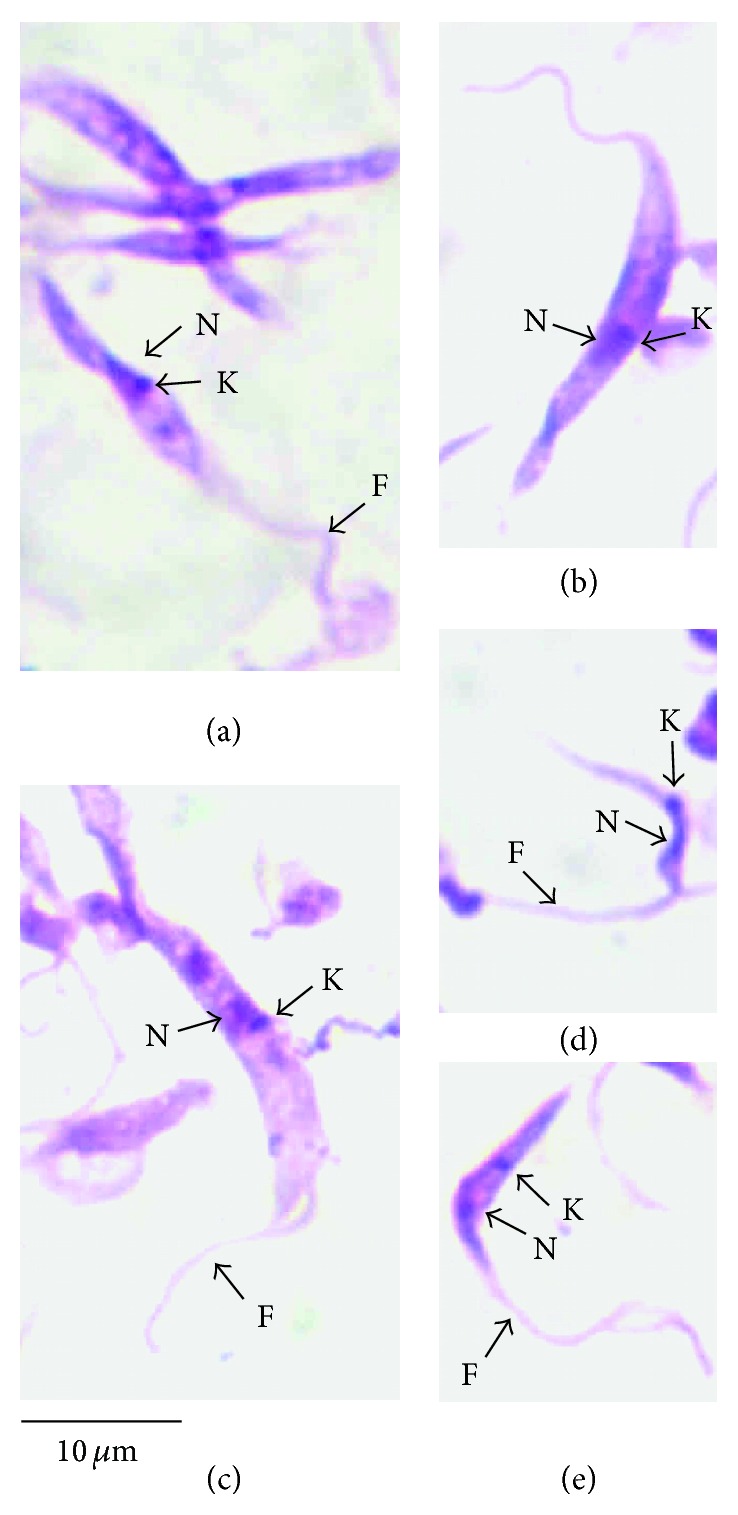
Microphotographs of Giemsa-stained trypanosome forms from *Cayman crocodilus *(CBT 02) obtained by in vitro culture (LIT). Epimastigotes forms (a, b, c) and trypomastigotes forms (d, e). K, kinetoplast; N, nucleus; F, flagellum.

The morphology consisted of epimastigotes with a large kinetoplast positioned near the nucleus and small and narrow undulating membrane (Figure 1) similar to the morphotype 1 in *C. yacare* trypanosomes previously described [19].

We determined sequences from gGAPDH and SSU rDNA (V7-V8 region) and these sequences were aligned with sequences from different trypanosomatids species retrieved from GenBank (Table 1).

**Figure 2:**
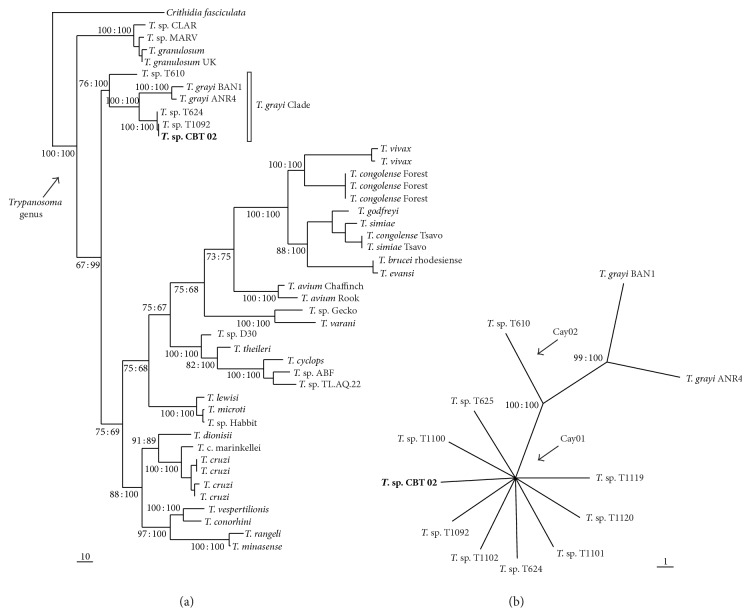
(a) Phylogenetic trees inferred by maximum parsimony and Bayesian methods based on gGAPDH gene sequences of 43 trypanosomes and *Crithidia fasciculata* as outgroup (515 characters, 34 parsimony informative). (b) Dendogram inferred by parsimony analyses based on V7-V8 SSU rDNA sequences from 12 crocodilian trypanosomes isolates (734 characters, 24 parsimony informative). Numbers at nodes show bootstrap (first) and posterior probabilities (second) values for MP and BI, respectively. The support values for the major branches are derived from 500 replicates, respectively, for MP.

Phylogenetic relationships based on gGAPDH and SSU rDNA sequences inferred by parsimony and Bayesian clustered this new isolate together to the major branch, which corresponds to crocodilian trypanosomes isolates (*T. grayi* Clade) (Figures 2(a) and 2(b)). The *T. grayi* Clade included both African crocodilians trypanosomes and American caymans trypanosomes. The isolates from Brazilian caimans were segregated into genotypes assigned Cay01 and Cay02 previously described [19].

Despite distinct host and different geographic origins, the isolate from *C. crocodilus* and Cay01 genotype from *C. yacare* shared high similarity (~0.1% for gGAPDH and identical SSU rDNA V7-V8 region). Phylogenetic analysis using the concatenated data set of gGAPDH and SSU rDNA genes generated very similar phylogenetic topologies (data not show).

## 4. Discussion

In Brazil only two trypanosomes have been reported in *Caiman* species based on morphological analysis of blood trypomastigote: unnamed species in *C. yacare* from Pantanal [22] and *Trypanosoma cecili* in *C. crocodilus* from Amazonia [13]. Recently, trypanosomes isolates were obtained from *C. yacare* in Pantanal from Mato Grosso do Sul State and showed similar morphology of blood trypomastigote with *Trypanosoma* sp. described previously [19, 22]. In this present study we obtained the first trypanosome isolate from *C. crocodilus*.

The prevalence of *Trypanosoma* sp. in *C. yacare* varies from 35 to 46% in different studies performed in Pantanal region from Mato Grosso do Sul state [19, 22]. Only one spectacled caiman captured in Mato Grosso was positive in hemoculture (33%), but the prevalence in this species has not been established and a further study involving a large number of individuals (*C. crocodilus*) is necessary to determine the prevalence of *Trypanosoma* sp. and *T. cecili*.

Most studies of caiman trypanosomes were performed in the Pantanal region. The only study in Amazon with *C. crocodilus* and *Paleosuchus trigonatus* found a very low prevalence with rare trypomastigote forms in blood or tissue. This species was designated *T. cecili* and isolation has not yet been possible [13].

The epimastigotes forms in axenic culture of spectacled caiman isolate are very similar to *Trypanosoma* sp. from *C. yacare* from Pantanal (Figure 1). The tissue imprint forms of *T. cecili* and blood forms of *Trypanosoma* sp. from Pantanal are distinguishable, but similar tissue forms are detected in *C. yacare* and *C. crocodilus* [13, 19, 22]. 

The variable V7-V8 region of SSU rDNA has been used for DNA barcoding of trypanosomatids and is able to distinguish all species, polymorphisms, and genetic relationships among closely related taxa [18, 19, 29–33]. In addition, the gGAPDH sequences were used to improve the phylogenetic analysis [19, 23, 24, 33]. In all phylogenetic analyses inferred by V7-V8 SSU rDNA and gGAPDH the isolated CBT 02 was included in monophyletic branch called *T. grayi* Clade, together with *Trypanosoma* sp. from Brazilian caiman. The V7-V8 region segregated the Brazilian caimans isolates in two branches: Cay01 and Cay02. Most isolates were comprised of Cay01 genotype, probably the most prevalent in Pantanal region. The new isolate from *C. crocodilus* (CBT 02) nested in Cay01 genotype. 

The phylogenetic positions of CBT02 isolate show that *Trypanosoma* sp. from Cay01 genotype I is able to infect two species of *Caiman* genus in Brazil. These findings clarify the morphological approaches in tissues imprints which have trypomastigote similar morphology in *C. yacare* and *C. crocodilus.*

The distribution of Cay01 genotype is not restricted to Pantanal region of Mato Grosso do Sul state and vertebrate host. The Confresa county is located in north of Mato Grosso state and comprises a mosaic of Amazonia and Cerrado biomes and is irrigated by Tocantins-Araguaia basin. The *C. yacare* occurred in upper Madeira system of the Amazon basin and Paraguay River and lower Paraná system including Brazil, Bolivia, Paraguay, and Argentina. *C. crocodiles* occur in both Amazon and Orinoco drainage [2, 5]. According to Brazaitis et al. [34], sympatric areas occur in Mato Grosso and Rondônia states. However, in Tocantins-Araguaia basin only *C. crocodilus* occurs.

The presence of the same trypanosome species in *C. yacare* and *C. crocodilus* indicates transmission cycles between these species evidencing sympatry of caiman species or exposure to the same vector. The *T. grayi* in Africa is transmitted by tse-tse flies [35]. In Brazil, different species of tabanids can bite reptiles like *C. crocodilus* and *Eunectes murinus* [36]. The low host specificity of tabanids and host switch of caiman trypanosomes suggest an important role of insects in the transmission cycle of these parasites in Brazil. 

The addition of new isolates of caiman trypanosomes of different vertebrate hosts is necessary for better understanding of diversity and phylogenetic relationships of these parasites and demonstrates the low host specificity of cayman trypanosomes in Brazil. 

## Figures and Tables

**Table 1:** Trypanosomatids isolates, host, geographic origin, and sequences of SSU rDNA and gGAPDH used for phylogenetic analysis.

Trypanosomatids species	Isolate code	Host	Geographic origin	Accession number^a^	
				gGAPDH	SSU rDNA
		Fish			
*Trypanosoma* sp.	CLAR	*Clarias angolensis *	Africa	AJ620251	
*Trypanosoma *sp.	MARV	*Cyprinus carpio *	Czech Republic	AJ620248	
*T. granulosum *	Portugal	*Anguilla anguilla *	Portugal	AJ620247	
*T. granulosum *	UK	*Anguilla anguilla *	United Kingdom	AJ620246	
		Reptile			
*Trypanosoma* sp.	Gecko	*Tarentola annularis *	Senegal	AJ620259	
*T. varani *	V54	*Varanus exanthematicus *	Senegal	AJ620261	
*Trypanosoma* sp.	T610	*Caiman yacare *	Brazil	EU596256	EU596252
*Trypanosoma* sp.	T624	*Caiman yacare *	Brazil	EU596257	EU596253
*Trypanosoma* sp.	T625	*Caiman yacare *	Brazil		EU596259
*Trypanosoma* sp.	T1092	*Caiman yacare *	Brazil	EU596258	EU596254
*Trypanosoma* sp.	T1100	*Caiman yacare *	Brazil		EU596260
*Trypanosoma* sp.	T1101	*Caiman yacare *	Brazil		EU596261
*Trypanosoma* sp.	T1102	*Caiman yacare *	Brazil		EU596262
*Trypanosoma* sp.	T1119	*Caiman yacare *	Brazil		EU596263
*Trypanosoma* sp.	T1120	*Caiman yacare *	Brazil		EU596255
*Trypanosoma* sp.	CBT02	*Caiman crocodilus *	Brazil	**JQ768791**	**JQ768792**
		Bird			
*T. avium *	Chaffinch	*Fringilla coelebs *	Czech Republic	AJ620263	
*T. avium *	Rook	*Corvus frugilegus *	Czech Republic	AJ620262	
		Mammals			
*T. conorhini *	USP	*Rattus rattus *	Brazil	AJ620267	
*T. cruzi marinkellei *	B7	*Phyllostomus hastatus *	Brazil	AJ620270	
*T. cruzi *				X52898	
*T. lewisi *	L32	*Rattus rattus *		AJ620272	
*T. microti *	TRL132	*Microtus agrestis *	England	AJ620273	
*Trypanosoma* sp.	ABF	*Wallabia bicolor *	Australia	AJ620276	
*Trypanosoma* sp.	R5	*Oryctolagus cuniculus *	Australia	AJ620276	
*T. theileri *	K127	*Bos taurus *	Germany	AJ620282	
*Trypanosoma *sp.	D30	Cervus dama	Germany	AJ620279	
*T. dionisii *	P3	Pipistrellus pipistrellus	United Kingdom	AJ620271	
*T. vespertilionis *	P14	Pipistrellus pipistrellus	England	AJ620283	
*T. brucei rhodesiense *	058	Homo sapiens	Zambia	AJ620284	
*T. vivax *	Desowitz	Ovis aries	Nigeria	AJ620295	
*T. vivax *				AF053744	
*T. congolense* Forest	Cam22	*Capra capra *	Cameroon	AJ620289	
*T. congolense* Forest	TSW103	*Sus scrofa *	Liberia	AJ620286	
*T. evansi *				AF053743	
*T. cyclops *	LV492	*Macaca* sp.	Malaysia	AJ620265	
*T. minasense *	LSTM	*Saimiri boliviensis *	South America	AJ620274	
*T. rangeli *				AF053742	
		Invertebrate			
*Crithidia fasciculata *				AF047493	
*T. congolense *Forest	ANR3	*Glossina palpalis *	Gambia	AJ620285	
*T. congolense* Tsavo	114	*Glossina pallidipes *	Tanzania	AJ620291	
*T. cruzi *	C8 cl2	*Triatoma infestans *	Bolivia	AJ620268	
*T. cruzi *	VINCH 89	*Triatoma infestans *	Chile	AJ620269	
*T. grayi *	ANR4	*Glossina palpalis *	Gambia	AJ620257	AJ005278
*T. grayi *	BAN1	*Glossina palpalis *	Gambia	AJ620258	AJ620546
*T. simiae *	Ken 2	*Glossina morsitans *	Gambia	AJ620293	
*T. godfreyi *	Ken 7	*Glossina morsitans *	Gambia	AJ620292	
*T. simiae *Tsavo	Ketri 1864	*Glossina pallidipes *	Kenya	AJ620294	
*Trypanosoma* sp.	TL.AQ.22	*Philaemon* sp.	Australia	AJ620280	

^a^Sequences determined in this study and deposited in GenBank are underlined and bold.
